# Integrating Machine Learning with Robotic Rehabilitation May Support Prediction of Recovery of the Upper Limb Motor Function in Stroke Survivors

**DOI:** 10.3390/brainsci14080759

**Published:** 2024-07-29

**Authors:** Sara Quattrocelli, Emanuele Francesco Russo, Maria Teresa Gatta, Serena Filoni, Raffaello Pellegrino, Leonardo Cangelmi, Daniela Cardone, Arcangelo Merla, David Perpetuini

**Affiliations:** 1 Department of Engineering and Geology, University “G. d’Annunzio” of Chieti-Pescara, 65127 Pescara, Italy; sara.quattrocelli@studenti.unich.it (S.Q.); leonardo.cangelmi@unich.it (L.C.); d.cardone@unich.it (D.C.); arcangelo.merla@unich.it (A.M.); 2 Padre Pio Foundation and Rehabilitation Centers, 71013 San Giovanni Rotondo, Italy; emanuele.russo@centripadrepio.it (E.F.R.); mariateresa.gatta@centripadrepio.it (M.T.G.); 3 I.R.R.C.S. Casa Sollievo della Sofferenza, 71013 San Giovanni Rotondo, Italy; s.filoni@operapadrepio.it; 4 Department of Scientific Research, Campus Ludes, Off-Campus Semmelweis University, 6912 Lugano-Pazzallo, Switzerland; raffaello.pellegrino@uniludes.ch; 5 Santa Chiara Institute, 73100 Lecce, Italy

**Keywords:** stroke, upper limbs, robotic rehabilitation, machine learning, Fugl-Meyer Assessment (FMA), Frenchay Arm Test (FAT), Barthel Index (BI)

## Abstract

Motor impairment is a common issue in stroke patients, often affecting the upper limbs. To this standpoint, robotic neurorehabilitation has shown to be highly effective for motor function recovery. Notably, Machine learning (ML) may be a powerful technique able to identify the optimal kind and intensity of rehabilitation treatments to maximize the outcomes. This retrospective observational research aims to assess the efficacy of robotic devices in facilitating the functional rehabilitation of upper limbs in stroke patients through ML models. Specifically, clinical scales, such as the Fugl-Meyer Assessment (A-D) (FMA), the Frenchay Arm Test (FAT), and the Barthel Index (BI), were used to assess the patients’ condition before and after robotic therapy. The values of these scales were predicted based on the patients’ clinical and demographic data obtained before the treatment. The findings showed that ML models have high accuracy in predicting the FMA, FAT, and BI, with R-squared (R^2^) values of 0.79, 0.57, and 0.74, respectively. The findings of this study suggest that integrating ML into robotic therapy may have the capacity to establish a personalized and streamlined clinical practice, leading to significant improvements in patients’ quality of life and the long-term sustainability of the healthcare system.

## 1. Introduction

Stroke is a sudden event caused by issues in blood flow to the brain, which can be ischemic (due to arterial occlusion) or hemorrhagic (caused by blood vessel rupture). Both types of stroke cause irreversible damage to brain tissue [1]. Ischemic stroke results from the occlusion of cerebral arteries, while hemorrhagic stroke results from vessel rupture in the brain [2,3,4,5].

This event is one of the leading causes of death and disability globally, with significant impacts on health and the economy [1]. Non-modifiable risk factors include age, gender, ethnicity, and genetics [6,7]. However, there are also modifiable factors such as hypertension, diabetes, smoking, and obesity that can be managed to reduce risk [7]. The incidence of stroke varies among different populations and geographical regions, with a global increase in recent decades [8,9,10]. Despite medical advancements, stroke remains a major cause of mortality and disability, resulting in substantial economic costs [11,12].

Notably, motor impairment in stroke patients is a prevalent and difficult problem that impacts a substantial proportion of people after a stroke. Studies suggest that around 80% of individuals who have had a stroke develop hemiplegia, which is a common and widespread consequence [13], and almost 70% of individuals who have had a stroke encounter functional impairments, with motor dysfunction being a prominent symptom [14]. This dysfunction is often linked to basal ganglia dysfunction [15], and alterations in the primary motor cortex (M1) [16]. Moreover, it is worth highlighting that upper limb recovery after a stroke is challenging, with only about 50% of stroke survivors likely to regain some functional use of their upper limbs [17]. This highlights the importance of effective interventions for upper limb rehabilitation post-stroke.

Various therapies have been examined to tackle motor impairment in individuals who have had a stroke. For instance, Transcranial Magnetic Stimulation (TMS) has been used to study motor dysfunction and recovery in stroke patients [18], and acupuncture targeting the basal ganglia has been demonstrated as effective in inducing neuroplasticity in stroke patients who have motor impairment [15].

In addition, therapies such as robotic neurorehabilitation have shown efficacy in the enhancement of motor functions of individuals with neurological disabilities [19]. Specifically, robotic neurorehabilitation represents an advanced form of therapy that uses robotic devices to assist patients during exercises and rehabilitation activities. These devices offer greater precision in movements and allow for more accurate customization of treatment based on the specific needs of the patient [20]. Additionally, robotic devices provide immediate and measurable feedback on recovery progress, encouraging and motivating patients during rehabilitation. This is particularly important, as highly motivated patients are more inclined to consider rehabilitation essential for recovery and to take an active role in the process [21,22]. They are also oriented towards independence and respond positively to information provided by professionals; this process facilitates learning and provides an objective assessment of patient performance, which are essential concepts in the rehabilitation journey [23]. Importantly, enhancing the patients’ attitude towards therapy, their awareness of the need for treatment, their need for prompts to participate, their level of active involvement in therapy activities, and their attendance throughout the rehabilitation program, is associated with reduced levels of depression, denial of disease, and negative emotional states, while also being connected to increased levels of positive emotional states [21].

Noteworthy, the employment of robotic rehabilitation for upper limbs in stroke patients has emerged as a promising approach to enhance motor recovery and functional outcomes post-stroke [24]. Among the robotic devices employed to this aim, the Armeo Power^®^ (Hocoma AG, Volketswil, Switzerland) has gained considerable interest in stroke rehabilitation because of its efficacy in improving the recovery of upper limb motor skills and functional outcomes in stroke patients. The Armeo Power is a specialized exoskeleton created to aid in the execution of complex upper limb movements in three dimensions. It offers rigorous and repeated training focused on the shoulder, elbow, wrist, and gripping motions, with the goal of enhancing motor skills and coordination [25]. This robotic device provides support for the whole upper limb, spanning from the shoulder to the hand. It is equipped with a gravity-support system that effectively offsets the weight of the patient’s arm during treatment sessions [26]. Studies have shown that the Armeo Power robot has a beneficial effect on the restoration of upper limb motor function in individuals who experienced a stroke [27].

However, it is worth to highlight that in order to maximize the outcomes of the rehabilitative process, it is crucial to define the appropriate dosage of robotic therapy. The need to understand which types and intensities of rehabilitation therapies result in optimal and cost-effective outcomes has been a driving force of research for decades [28,29,30]. However, maximizing rehabilitation outcomes requires a thorough understanding of how dosage, frequency, and type of therapy influence recovery. In this context, the use of machine learning (ML) can be a powerful tool. In fact, through the analysis of a wide range of clinical and individual data, ML can help identify which rehabilitation therapies are most effective for each patient, thereby contributing to maximizing outcomes and improving quality of life after a stroke [31,32]. Specifically, recent studies have employed ML algorithms to classify stroke patients and healthy controls, analyzing parameters such as gait and electromyography (EMG) [31]; other ML models have demonstrated significant accuracy in diagnosis, risk stratification, optimizing medical treatment, and predicting patient prognosis [32]. Moreover, ML models have also been utilized to adjust the difficulty of a controller in the unstable training mode of a trunk rehabilitation robot, with the goal of optimizing participants’ balance performance and postural stability [33] and to predict improvements in daily activities and patient participation after stroke intervention by analyzing demographic data, stroke characteristics, and initial assessment scores [34]. Additionally, ML was used to automatically analyze patients’ muscle activity during walking videos, thereby enhancing the evaluation and support for rehabilitation [35].

This retrospective observational study aims to evaluate the effectiveness of robotic devices in the functional recovery of upper limbs in stroke patients. The main objective is to analyze the progress made by patients during robot-assisted rehabilitation and to develop ML models to predict such improvements. Several ML approaches have been investigated in detail to predict clinical metrics employed to assess upper limb motor function after the robotic rehabilitation, including the Fugl-Meyer Assessment (A-D) (FMA) [36], the Frenchay Arm Test (FAT) [37], and the Barthel Index (BI) [38], starting from demographic and clinical data measured before the robotic treatment.

## 2. Materials and Methods

### 2.1. Participants

In the context of this retrospective study, a sample of 30 acute stroke patients was considered; among the participants, there were 16 males and 14 females, with an average age of 64.6 years old (standard deviation: 14.9), and it was found that the stroke affected the dominant limb of 14 participants. The inclusion criteria for this study are as follows: individuals between the ages of 18 and 85 years old, who have experienced their first-ever ischemic stroke as confirmed by brain imaging (CT or MRI). Participants must also have severe or moderate upper limb hemiparesis, as determined by their FMA score (FMA ≤ 44). The stroke should be in the subacute phase, occurring within 60 days from the onset. Additionally, participants should have a Modified Ashworth Scale (MAS) score of less than 3 for the shoulder, elbow, and wrist.

Exclusion criteria for this study include stroke occurring in the brain stem or cerebellum, unstable general clinical conditions, severe visual impairment, recent or planned upper limb botox injections, orthopedic or neurological diseases affecting the function of the affected upper limb, discontinuation of treatments for at least 1 week or 5 consecutive sessions, and participation in other innovative treatment protocols for the upper limb.

### 2.2. Clinical Evaluation

To assess upper limb motor function in stroke patients, in this study the FMA, FAT, and BI were employed. The FMA is a widely validated procedure usually employed to evaluate body function according to the International Classification of Functioning, Disability, and Health (ICF) [36]. The FMA is grounded in a hierarchical understanding of motor recovery post-stroke, reflecting the natural sequence of motor return and sensory restoration. The FMA comprises several domains assessing different facets of sensorimotor function:

1. Motor Function: this domain evaluates voluntary movement within hemiplegic limbs and includes assessments of reflex activity, voluntary movements within synergistic patterns, and movements that progress beyond these synergies. It is scored using a three-point ordinal scale, where 0 indicates no movement, 1 partial movement, and 2 complete movement within normal limits.
2. Sensory Function: the sensory evaluation measures light touch and proprioceptive acuity, crucial for effective motor control.
3. Balance: this component assesses static and dynamic balance capabilities, integral for functional independence.
4. Joint Function: this includes assessments of range of motion and joint pain, which are vital for determining the mechanical constraints on voluntary movement and potential sources of discomfort.
5. Coordination and Speed: these tests evaluate the ability to perform movements involving coordination and speed, aspects that are often compromised following neurological damage.

Each section is scored separately, and the sum provides a comprehensive measure of an individual’s sensorimotor abilities. The maximal scores differ based on whether the entire assessment or specific sections are administered, facilitating flexibility in clinical and research settings. The FMA is highly regarded not only for its thoroughness but also for its ability to provide objective measures for tracking rehabilitation progress and evaluating the efficacy of interventions. This standardization makes the FMA a cornerstone in stroke rehabilitation research and practice, providing consistent, reproducible results across different clinical environments. Importantly, in this study, only the motor performance items of the upper extremity (0–66) were considered.

The FAT measures proximal motor control of the upper extremity and dexterity during activities of daily living (ADL) [37]. Developed to provide a straightforward and reliable measure of arm function, the FAT specifically focuses on the assessment of arm and hand movements that are essential for performing daily activities. The FAT consists of five tasks, each scored on a binary scale where a score of 1 indicates successful completion and a score of 0 indicates failure. The tasks assessed include the following:

1. Reaching and Retrieving: the patient is asked to reach out and pick up a small wooden block, testing the ability to coordinate movements of the shoulder and elbow.
2. Gripping and Holding: the patient must grasp the same block with a full hand grip, assessing the basic handgrip functionality.
3. Voluntary Movement of the Hand to the Back of the Head: this task examines the range of motion and dexterity required to perform more complex, coordinated movements.
4. Voluntary Movement of the Hand to the Mouth: the patient must touch their mouth, evaluating the ability to perform essential daily activities like eating.
5. Placing the Hand onto the Opposite Shoulder: this part of the test assesses the patient’s ability to cross the midline of the body, which is crucial for a range of daily tasks.

Each task is designed to mimic practical, everyday activities, thereby assessing the practical motor capabilities of the upper extremities. The total score is the sum of the scores from the five tasks, with a maximum score of 5 indicating full function.

Lastly, the BI serves as a simple measure of independence to assess rehabilitation progress; it comprises 10 tasks evaluated based on the time or assistance required by the patient [38]. It quantitatively assesses a person’s degree of independence in ten areas of ADL and mobility. The BI has been extensively used in clinical settings and research to evaluate the functional independence of patients, particularly in the contexts of rehabilitation following stroke, neurological disorders, and elderly care.

The BI focuses on ten specific tasks:

1. Feeding: to assess the ability to eat independently using appropriate utensils.
2. Bathing: to evaluate the ability to wash oneself in the bath or shower.
3. Grooming: to include tasks such as face washing, shaving, and teeth brushing.
4. Dressing: to measure the ability to select and wear clothes and shoes.
5. Bowels: to assess control over bowel movements.
6. Bladder: to evaluate control over bladder function.
7. Toilet Use: to measure the ability to get to the toilet, use it appropriately, and return independently.
8. Transfers: to include movements such as from wheelchair to bed and return, including sitting up in bed.
9. Mobility: to assess walking on a level surface or using a wheelchair over a designated distance.
10. Stairs: to evaluates the ability to ascend and descend stairs independently.

Each task is scored on a scale, typically 0, 5, 10, or 15, indicating the level of assistance required. The total possible score is 100, with higher scores representing greater independence. The scale is designed to be simple and quick to administer, requiring only a short time to complete through direct observation and interview. The BI is particularly noted for its reliability and validity in various patient populations. It is sensitive enough to detect clinical changes over time, making it valuable for monitoring progress in rehabilitation settings. Furthermore, the index’s focus on basic physical ADLs relevant to everyday living contributes to its widespread use in predicting the need for support services upon discharge from hospital or rehabilitation facilities.

Patients participated in a rehabilitation program consisting of 20 sessions, each lasting 50 min. The sessions were carried out 5 times per week and included the use of the Armeo Power exoskeleton. Additionally, patients received 1 session of traditional rehabilitation therapy that lasted the same period.

The Armeo Power is a motorized orthosis designed for the upper limb, offering six degrees of freedom (DoFs). These include three DoFs for the shoulder, one for elbow flexion, one for forearm supination, and one for wrist flexion. Every joint is motorized and fitted with two angle sensors. The robot can bear the weight of the patient’s arm, creating a sense of movement, and aiding in a wide range of three-dimensional movements during the execution of exercises. The use of a suspension system enables the operator to establish and modify the responsiveness of the robot based on the individual attributes of each patient. The lengths of the arm and forearm may be adjusted to accommodate a wide range of patients. The interface used for the implementation of workouts, presented in the form of games, is specifically developed to replicate arm movements and provide a straightforward virtual setting. Users may choose from several degrees of difficulty, which will decide the speed, direction, and range of movement based on the individual’s mobility throughout recovery.

Each robotic session was divided into two parts: the first part, lasting 10 min, involved passive mobilization to familiarize the patient with the exercises and reduce any spasticity they may have, whereas the second part, lasting 40 min, consisted of task-oriented exercises that were adjusted based on the patient’s abilities. The difficulty of these exercises gradually increased throughout the training period.

### 2.3. Experimental Design

The study was designed as an observational retrospective clinical study conducted on a cohort of patients affected by acute stroke. Detailed demographic and clinical data were collected, including age, sex, side of hemiplegia, dominant arm, and Bamford classification (i.e., Total anterior circulation infarct, TACI, Posterior circulation infarct, POCI, Lacunar infarct, LACI, Partial anterior circulation infarct, PACI), along with the time elapsed since the initial event [39]. Differences in the performance of the affected limb’s movement were evaluated using the FMA, FAT, and BI both at T0, before the start of the treatment, and at T1, one month after the treatment initiation, to monitor the rehabilitation progress over time. Notably, clinical information after 15 days from the first robotic session are available for 12 patients of the study sample. This approach allowed for a detailed characterization of the study population and assessment of the treatment effectiveness in relation to individual variables.

### 2.4. Data Analysis and Machine Learning

The data analysis, model implementation, training, and testing were conducted using MATLAB 2023b. Particularly, paired *t*-tests were employed to compare the scores of the tests employed at T0 and T1. Additionally, a repeated measure analysis of variance (RM-ANOVA) was performed to compare the clinical scores of those patients whose data after 15 days were available, followed by a post hoc analysis with multiple comparisons and Bonferroni correction.

Notably, the normality of the distribution was assessed through the Jarque–Bera test and visual inspection of normal probability plots. This approach was chosen to compare differences between two measurements obtained on the same group of individuals at different time points, eliminating inter-individual variability. Cohen’s d coefficient was calculated to assess the effect size of these changes, providing information on the clinical relevance of changes over time in motor and functional abilities of participants.

Additionally, ML models were implemented to predict outcomes following rehabilitation. These models used a dataset comprising demographic, clinical, and functional data collected from patients, along with FAT, FMA, and BI measurements, to predict future rehabilitation outcomes. Regarding demographic data, several variables were considered, including patients’ age. Specifically, age was categorized into four groups: less than 30 years, 31–50 years, 51–70 years, and over 70 years, to assess how age might influence rehabilitation response. Another demographic variable considered was patients’ gender, which may have implications on rehabilitation response and thus was included in the models.

Regarding clinical data, several variables were considered, including patients’ dominant arm, side of hemiplegia (i.e., the side of the body affected by stroke), time elapsed from stroke event to start of rehabilitation, and Bamford classification of stroke. These clinical factors can significantly influence prognosis and rehabilitation response. Finally, functional data included FAT, FMA, and BI measurements at T0, which were used as indicators of patients’ motor and functional abilities.

The combined use of all these data in ML models allowed identification of which variables were most significant in predicting rehabilitation effectiveness and what could be the expected outcomes for individual patients.

The first model trained was a bagging ensemble of decision trees; this approach involves creating multiple decision trees, each trained on a random subset of the training data. In this case, a set of decision trees with a minimum leaf size set to 8 and considering all available variables during training were utilized. Bagging randomly selected a subset of the training data for each tree, repeating the process for 30 learning cycles, ensuring sufficient diversity among trees in the ensemble. This improved the model’s ability to capture various nuances of the relationship between predictor variables and the response variable without overly fitting to the training data.

The second model trained was a Gaussian process regression (GP), a technique that uses a Gaussian process to model the relationship between predictor variables and the response variable. In this case, GP was defined by a kernel function, determining the shape of the relationship between variables. Different model parameters were specified, including the constant base function and the exponential kernel function. The GP regression was trained using the training data, aiming to find the best representation of the GP that fits the observed data. This process involves estimating model parameters, including the kernel, to maximize the likelihood of the observed data.

The third model trained was a kernel regression, which uses a kernel function to model the relationship between predictor variables and the response variable. Regression is performed using the least squares method, which seeks to minimize the sum of squares of differences between model predictions and actual values of the response variable. Several parameters were considered in the model setup, such as the number of kernel expansion dimensions, the regularization parameter Lambda, and the kernel scale. Lambda regulates the importance of regularization terms in the model, controlling its complexity; the parameters were automatically optimized during training to maximize the model’s predictive ability.

The fourth model trained was a neural network for regression, a type of model that uses a neural network structure to predict the response variable based on the provided predictor variables. In the model configuration, the neural network was defined with a hidden layer containing 100 neurons and using the Rectified Linear Unit activation function with a maximum number of iterations set to 1000.

All models underwent leave-one-out cross-validation, with Root Mean Square Error (RMSE), Mean Square Error (MSE), and R squared (R^2^) used as evaluation metrics. In order to assess the consistency between the measured variables and the predicted ones, paired *t*-tests, regression line graphs, and Bland–Altman plots were employed.

## 3. Results

From the Jarque–Bera test, it emerged that the data follow a normal distribution. The results include the *p*-values and h-stat in Table 1.

**Figure 1:**
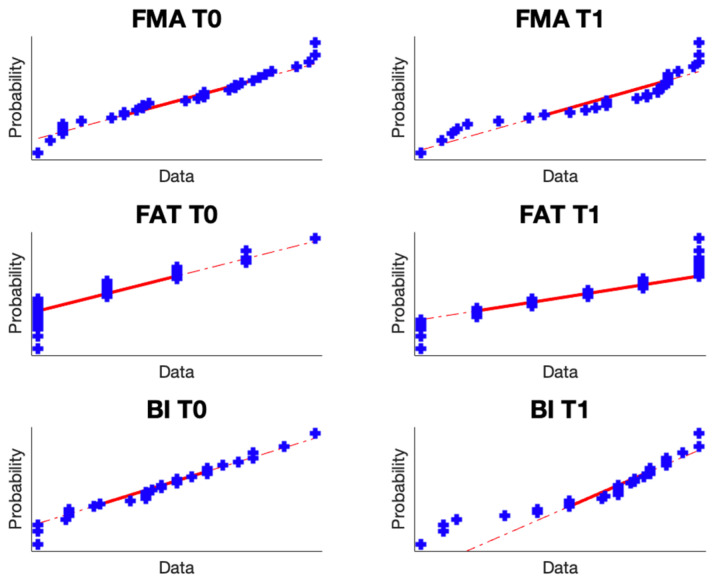
Normplot of the data.

Additionally, a normplot was conducted to further confirm the normality of the data, as shown in Figure 1.

Significant changes were observed between T0 and T1 in the evaluation of clinical scales. The *t*-test revealed a significant difference for FMA, as well as for FAT and BI, as reported in Table 2.

Additionally, to assess the effect size between T0 and T1, Cohen’s d indices were calculated. For FMA, the Cohen’s d index was 1.733, for FAT it was 1.302, and for BI it was 1.424. Furthermore, the RM-ANOVA performed for those patients whose data were available also after 15 days from the first administration delivered *p*-values for FMA, FAT, and BI between pre-treatment, 15 days, and 1 month of 7.876 × 10^−4^, 2.285 × 10^−2^, and 2.161 × 10^−2^, respectively. The *p*-values of the *t*-tests for FMA related to multiple comparisons between pre-treatment and 1 month, pre-treatment and 15 days, and 15 days and 1 month were, respectively, 2.569 × 10^−7^, 1.057 × 10^−5^, and 7.373 × 10^−4^. For FAT, the *p*-value values related to multiple comparisons between pre-treatment and 1 month, pre-treatment and 15 days, and 15 days and 1 month were 2.168 × 10^−3^, 2.010 × 10^−3^, and 2.098 × 10^−2^. Concerning the BI, the *p*-value values related to multiple comparisons between pre-treatment and 1 month, pre-treatment and 15 days, and 15 days and 1 month were 2.168 × 10^−3^, 2.168 × 10^−3^, and 1.271 × 10^−3^. Moreover, a *t*-test was conducted to determine if there were differences in mobility recovery at 1-month post-treatment, considering whether the affected limb was the dominant or non-dominant one. The results of the *t*-tests for FMA, FAT, and BI between dominant and non-dominant arm are as follows: the *p*-value for FMA is 0.027, for FAT is 0.096, and for BI is 0.018.

**Figure 2:**
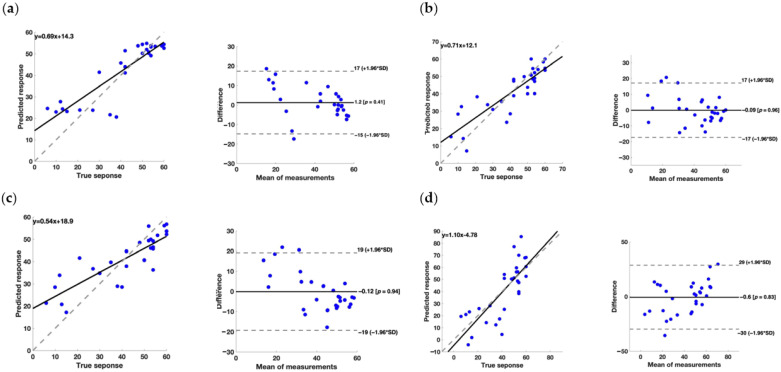
(**a**) On the left, the regression line plot, and on the right, the Bland–Altman plot for the FMA score of the Bagged Ensemble of Decision Trees model. (**b**) On the left, the regression line plot, and on the right, the Bland–Altman plot for the FMA score of the Gaussian Process model. (**c**) On the left, the regression line plot, and on the right, the Bland–Altman plot for the FMA score of the Kernel Regression model. (**d**) On the left, the regression line plot, and on the right, the Bland–Altman plot for the FMA score of the Neural Network model.

**Figure 3:**
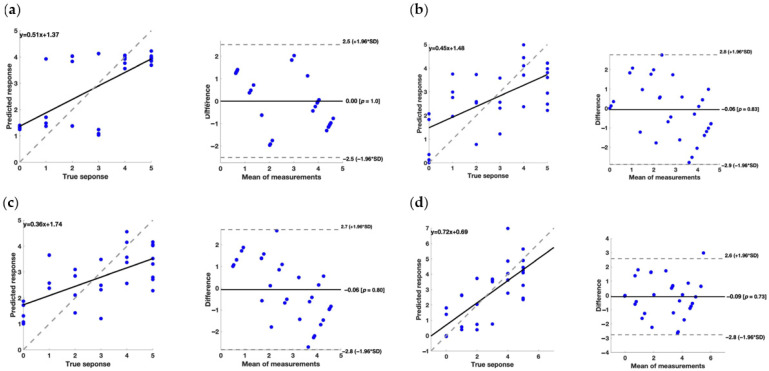
(**a**) On the left, the regression line plot, and on the right, the Bland–Altman plot for the FAT score of the Bagged Ensemble of Decision Trees model. (**b**) On the left, the regression line plot, and on the right, the Bland–Altman plot for the FAT score of the Gaussian Process model. (**c**) On the left, the regression line plot, and on the right, the Bland–Altman plot for the FAT score of the Kernel Regression model. (**d**) On the left, the regression line plot, and on the right, the Bland–Altman plot for the FAT score of the Neural Network model.

**Figure 4:**
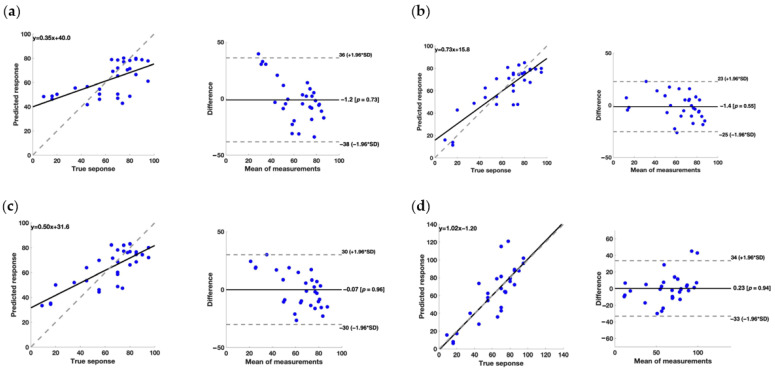
(**a**) On the left, the regression line plot, and on the right, the Bland–Altman plot for the BI score of the Bagged Ensemble of Decision Trees model. (**b**) On the left, the regression line plot, and on the right, the Bland–Altman plot for the BI score of the Gaussian Process model. (**c**) On the left, the regression line plot, and on the right, the Bland–Altman plot for the BI score of the Kernel Regression model. (**d**) On the left, the regression line plot, and on the right, the Bland–Altman plot for the BI score of the Neural Network model.

To assess the effectiveness of the models, we examined the regression line plots and Bland–Altman plots; these results are presented in Figure 2, Figure 3 and Figure 4 each showing the various scores considered in the analysis.

The results are summarized in Table 3.

Afterwards, *t*-tests were conducted between the true values and the predicted values by the model to assess the significance of the predictions; the obtained *p*-values are reported in Table 4.

## 4. Discussion

The main objective of the research was to leverage the potential of ML to predict clinical outcomes related to robotic assistive therapy for the upper limbs’ recovery in post-stroke patients. To this aim, various significant parameters were considered, including patients’ age, gender, distance from the stroke event, and the onset of rehabilitation, along with the Bamford classification to define the type of stroke and whether the affected arm was dominant or not. This allowed correlations and patterns that could positively influence the rehabilitation process to be identified.

The results of the inferential analysis using the *t*-tests revealed significant differences between the values of FAT, FMA, and BI scores across the two time points (T0 and T1). This result suggests that the treatment significantly improved the motor functions of the patients. Additionally, to assess the effect between T0 and T1, Cohen’s d indices were calculated. The obtained values indicate a medium to large effect of the treatment on all three clinical scales, with FMA showing the largest effect, followed by BI, and finally FAT. This suggests that the treatment had a significant impact on patients’ mobility, strength, and daily activities scores.

For 12 subjects, FMA, FAT, and BI values were also available midway between T0 and T1, at 15 days post-treatment. The RM-ANOVA, followed by a post hoc analysis with Bonferroni correction, revealed significant differences between the three time periods for each clinical scale, with an improvement observed over time. Finally, a *t*-test was conducted to determine if there were differences in mobility recovery at one-month post-treatment, considering whether the affected limb was the dominant or non-dominant one. The results show that the differences between dominant and non-dominant arms are statistically significant for FMA and BI, while they are not significant for FAT. These results suggest that the treatment’s effect on mobility recovery may vary depending on the involved limb.

For the FMA clinical scale, the Bagged Ensemble of Decision Trees model demonstrated a good level of fit to the data, with a coefficient of determination (R^2^ validation) of 0.79. However, the RMSE validation (0.368) and MSE Validation (0.226) values are moderately high, suggesting that the model’s predictions may have some degree of error.

Regarding the Gaussian Process model, it achieved a slightly lower R^2^ validation (0.73) compared to the Bagged Ensemble of Decision Trees model, but the RMSE validation (0.393) and MSE Validation (0.261) values are similar; this indicates that, despite the model’s ability to adequately explain the variation in the data, its predictions may be as accurate as the decision tree-based model. The results suggest that the Bagged Ensemble of Decision Trees model may be the best choice to predict the FMA clinical scale, thanks to its good balance between data adaptability and prediction accuracy. Notably, the Gaussian Process model also offers a valid alternative with similar results.

For the FAT clinical scale, the obtained results are influenced by the fact that it is a reduced scale; therefore, even small discrepancies in the predicted values can be significant considering the narrow range of values of the scale. The Bagged Ensemble of Decision Trees model achieved an R^2^ of 0.49, indicating that approximately 49% of the variation in the data can be explained by the predictor variables used; however, the RMSE validation (0.591) and MSE Validation (0.482) values are quite high, suggesting that the model’s predictions may have a significant degree of error, even considering the reduced scale of the target variable. Notably, the Kernel Regression model showed an R^2^ of 0.46, with RMSE validation (0.696) and MSE Validation (0.633) values higher than the Bagged Ensemble of Decision Trees, suggesting that the predictions of the Kernel Regression model may be less precise than the first model. Therefore, for the FAT scale, the results of the models are influenced by its reduced nature, with small discrepancies in the predicted values that can have a significant impact. In fact, a reduced range is associated with a limited variability for the model to learn from. This can make it harder for the model to distinguish between the different values and make accurate predictions. Notably, this aspect is intrinsic to the learning process of the machineries, but it does not invalidate the reliability of the results. The Bagged Ensemble of Decision Trees model appears to be the best choice in terms of balance between data adaptability and prediction accuracy, even considering the sensitivity of the FAT scale

For the BI clinical scale, the Gaussian Process model achieved the highest R^2^ (0.74), indicating better data adaptability; however, the RMSE validation (0.416) and MSE Validation (0.255) values are lower when compared to the other models, suggesting that the model’s predictions may be more accurate compared to the others. Hence, among all the models considered, the Gaussian Process model seems to offer the best balance between data adaptability and prediction accuracy.

From the statistical analysis conducted through the *t*-tests between the true values and those predicted by the four models (Bagged Ensemble of Decision Trees, Gaussian Process, Kernel Regression, and Neural Network), it emerged that the *p*-values are all greater than 0.05; this result indicates that there are no statistically significant differences between the true values and those predicted for the three clinical scales under consideration. These results demonstrate that no-bias is present between the measured and the predicted values of all the metrics considered, showing good performance of the models.

Considering the Bland–Altman plot, it should be highlighted that some models presented a systematic error in the prediction of the rehabilitative metrics. Particularly, concerning the FMA, the error associated with the prediction performed through the Kernel Regression model showed a negative slope, whereas the Neural Network model had a positive trend. Regarding the FAT, the prediction performed through the Kernel Regression model exhibited a negative relationship between the error and the value of the metric. For the BI, the Bagged Ensemble of Decision Trees model and the Kernel Regression model exhibited a trend in the errors related to the value of the BI. These results are also confirmed by the regression equation linking the predicted and measured metrics. In fact, a model should present a slope of 1 and a quote of 0 to perfectly represent the measured values. Concerning the FMA, the Neural Network model provided the slope closest to 1, but the quote is quite far from 0 (−4.78). Also, regarding the FAT and BI, the best result was achieved by the Neural Network model, with a slope of 0.72 and 1.20, and a quote of 0.69 and −1.20, respectively.

Importantly, it should be stressed that for the choice of the best model, the presence of biases and systematic errors and the slope and quote of the regression should be taken into account. Particularly, concerning the systematic errors, they can be mathematically corrected which will improve the performance of the models. Considering the strength and the weakness of each model developed, it is clear that further studies are needed for a refinement of the performance.

It should be considered that the analysis was conducted on a relatively limited sample of subjects, which may limit the generalizability of the results; future studies with larger samples could confirm these conclusions. Importantly, despite the study’s relatively small sample size, the investigation utilized a leave-one-out cross-validation procedure. This involved removing one subject at a time and testing the regressor outcome on that subject, allowing for an evaluation of the out-of-sample performance, improving the generalizability of the findings. Expanding the sample size might potentially enhance the effectiveness of the classifier by mitigating the risk of overfitting within the sample. Furthermore, increasing the sample size will allow for employing more advanced regression algorithms (e.g., Deep Learning), which were not used in this study because of the reduced sample numerosity and the probable over-fitting effect. Moreover, it should be highlighted that the results rely on retrospective observational data, thus prospective or randomized controlled trials should be performed in order to confirm the generalizability of the findings, the long-term efficacy, and cost-effectiveness. In fact, further studies will allow for a more frequent data collection (not limited to two or three sessions in one month), providing a better understanding of the rehabilitation progress. Additionally, most subjects in the sample fell within the age range of 51 to 70 years, which may limit the ability to generalize the results to different age groups. However, it should be noted that previous studies reported that the incidence of stroke peaks in the age range of 46–65 years [40], that the vast majority of stroke victims are over the age of 60 [41], and that the average age of stroke survivors is around 64–65 years [42]. These findings suggest that the age range considered in this study is indicative of the age most affected by stroke, but enlarging the dataset will allow for the consideration of other age ranges, thus increasing the generalizability of the models. Additionally, it is crucial to highlight that SP can exhibit variability in their recovery trajectories, hence it is crucial to consider the variability intrinsic to the population in the training sample, in order to make the model able to take into account this variability for the prediction. Indeed, tailoring models for each patient can provide more detailed prediction outcomes, but models, as those developed in this study, able to provide a prediction of the recovery considering the demographic, clinical, and functional data collected from patients, may be a source of support for the clinicians for planning the rehabilitation therapy. In this perspective, further studies should be implemented to consider other scales able to capture all dimensions of motor function recovery or patient well-being.

Another aspect to consider is that the data were collected using assessment tools, which may introduce a risk of error due to subjectivity or limited precision of the instruments used. Other factors that could influence rehabilitative outcomes, such as overall health status or adherence to therapies, could be considered. In fact, a patient’s general health and consistent adherence to rehabilitation protocols and therapies is crucial for achieving optimal results. Ensuring that patients are motivated and supported in following their treatment plans can enhance the effectiveness of rehabilitation efforts. For this reason, further studies should be performed taking into account this aspect in the ML models.

However, in this context, it is pertinent to note that several previous studies have focused on fundamental aspects in the predictive and therapeutic field using advanced machine learning techniques. A previous study explored the use of various ML algorithms to improve the accuracy of predicting upper limb motor recovery by integrating clinical data with detailed biomechanical measurements. Specifically, the study utilized the Motor Activity Log (MAL) to measure the activity of the paretic arm at home and combined these data with upper limb kinematics during reaching and grasping tasks measured in the laboratory. The results revealed significant variations in prediction accuracy depending on the combination of features and algorithms used: for predicting the MAL ratio, using two features related to trunk compensation with the KNN algorithm achieved an accuracy of 84.4% before applying Principal Component Analysis (PCA); using two features from ARAT and Fugl-Meyer scores with the SVM algorithm increased the accuracy to 86.6% without applying PCA; the best result for predicting the MAL ratio, with an accuracy of 93.3%, was achieved by combining three features from ARAT and trunk compensation with the Random Forest algorithm after applying PCA. For predicting the accel-ratio, using two features related to trunk compensation with the SVM algorithm resulted in an accuracy of 77.7% before PCA; using one feature from either ARAT or Fugl-Meyer with Birch or KNN algorithms improved the accuracy to 84.4% without PCA; the most accurate prediction for the Accel ratio, with an accuracy of 88.8%, was obtained by combining three features from ARAT and trunk compensation with the KNN algorithm after applying PCA [43]. Another study employed Logistic Regression, Random Forest, and Decision Tree algorithms to predict the risk of stroke, taking into account the influence of smoking habits and optimizing healthcare management strategies for affected patients. The results showed that the Decision Tree, with smoking status included, achieved an accuracy of 98.78%, which increased to 99.46% without considering smoking status; meanwhile, Logistic Regression reached an accuracy of 71.21% with smoking status and 81.34% without; Random Forest, on the other hand, achieved an impressive accuracy of 99.98% including smoking status [44]. Another approach examined a broad set of variables, including demographic data, initial National Institutes of Health Stroke Scale scores, and laboratory test results, using deep neural networks, Random Forests, and Logistic Regression to develop predictive models for post-stroke outcomes. This study included a total of 2,604 patients, of whom 2043 (78%) had favorable outcomes. The developed predictive models were compared with the ASTRAL score, an assessment system used to estimate risk and predict post-stroke outcomes based on various clinical and demographic factors. The area under the curve (AUC) for the deep neural network model was significantly higher than that of the ASTRAL score (0.888 vs. 0.839; *p* < 0.001); however, the areas under the curves for the Random Forest (0.857; *p* = 0.136) and Logistic Regression (0.849; *p* = 0.413) models were not significantly higher than that of the ASTRAL score [45]. Also, the use of Decision Trees, Naïve Bayes, and Artificial Neural Networks was investigated to accurately identify patients at risk of stroke. These models demonstrated varying levels of performance, with Decision Trees providing clear interpretability and good accuracy, Naïve Bayes offering simplicity and effectiveness with a solid performance, and Artificial Neural Networks achieving the highest accuracy due to their ability to capture complex patterns in the data: the results led to the proposal of personalized preventive interventions through a dedicated web platform for health management, aiming to enhance early detection and prompt lifestyle modifications [46]. Another study demonstrated the effectiveness of a weighted voting approach, combining ten different classifiers for stroke prediction and early detection; the classifiers used in this approach are Logistic Regression, Stochastic Gradient Descent, Decision Tree Classifier, AdaBoost Classifier, Gaussian Classifier, Quadratic Discriminant Analysis, Multi-layer Perceptron Classifier, K-Nearest Neighbors Classifier, Gradient Boosting Classifier, and XGBoost Classifier. The results of the base classifiers are aggregated using the weighted voting approach to achieve the highest accuracy, reaching an accuracy of 97% [47].

Moreover, a multimodal approach should be implemented, hence further studies are indeed necessary to evaluate, for instance, the neuroplasticity induced by the robotic rehabilitation of the upper limbs in stroke patients through the employment of portable neuroimaging tools [48,49]. To this aim, further studies including, for instance, electroencephalography (EEG) or functional near infrared spectroscopy (fNIRS) could provide further information regarding the effect of the rehabilitation to the neural plasticity. Importantly, it should be highlighted that the quality and completeness of clinical and demographic data are critical to the accuracy and robustness of ML models in healthcare. High-quality data ensures accurate representation of real-world scenarios, preventing errors and inconsistencies that could degrade model performance. Completeness of data is equally important, as handling missing data appropriately and ensuring comprehensive coverage prevent biases and enhance model generalization. Robust ML models require high-quality and complete data to generalize well to new data, avoiding overfitting and underfitting. In this study, a reliable validation and testing of models was performed thanks to the quality and completeness of the data, ensuring accurate performance metrics.

Concerning the employment of the developed models in clinical practice, it should be considered that they take as input demographic, clinical, and functional data collected from patients that are already considered in the clinical workflows. Hence, employing these models implies the development of a software with a user-friendly graphical user interface where the clinicians can provide as input the requested information already acquired during the clinical assessment. To this aim, training sessions for healthcare professionals can be organized to properly employ these models in clinical practice.

It should be highlighted that the use of ML opens broad prospects in the field of rehabilitation, allowing for personalized treatment tailored to each patient and for constant monitoring of patient progress, thereby adjusting therapies in real time. The employment of the developed method ensures a more effective and targeted treatment, while simultaneously reducing the risk of complications or therapeutic inefficacy. Therefore, ML not only provides new opportunities to optimize rehabilitative outcomes but also to identify and address potential obstacles to patient progress early on. Importantly, these results could also foster the establishment of shared guidelines concerning the administration of robotic rehabilitative therapies for the upper limbs recovery. In this perspective, it is worth noting that although establishing shared guidelines for robotic rehabilitative therapies can standardize clinical practices and improve outcomes, achieving this requires extensive validation and collaboration. Rigorous clinical trials and evidence-based research are necessary to ensure guidelines are effective across diverse settings. Moreover, collaboration among clinicians, researchers, technology developers, and policymakers is crucial, alongside centralized data sharing to continuously update guidelines. Additionally, training and support for healthcare providers are also essential for effective implementation. Thus, while promising, realizing these guidelines will need comprehensive validation and cooperative efforts across the healthcare spectrum.

In addition, ethical and privacy implications of the employment of ML in clinical practice should be considered. In fact, the use of physiological and rehabilitative data presents major issues about user privacy and autonomy. Hence, establishing a framework for ethical monitoring procedures is essential; however, defining widely acceptable rules remains an ongoing issue. In this perspective, another ethical issue is related to the fact that advanced technologies like robotic devices and ML could make the rehabilitation process less accessible, especially in resource-limited settings. To address this issue, several strategies can be implemented. Cost reduction can be achieved through subsidies, grants, and bulk purchasing, making technologies more affordable. In addition, training programs and tele-rehabilitation can ensure that local healthcare providers are equipped to use these technologies effectively. Furthermore, partnerships between the public and private sectors, as well as international cooperation, can facilitate the sharing of resources and knowledge. However, defining a solution to this relevant issue is still an ongoing concern. Finally, it should be highlighted that integrating ML in robotic therapy could pave the way for a more personalized and effective clinical practice, with a significant impact on patients’ quality of life and the sustainability of the healthcare system.

## 5. Conclusions

This research focused on using ML to maximize robotic rehabilitation outcomes in stroke patients for the recovery of the upper limbs’ motor functions. The results indicate that employing ML can significantly optimize rehabilitation outcomes, allowing successful prediction of rehabilitative metrics (i.e., FMA, FAT, and BI) from pre-therapy values. This approach will foster the creation of standardized protocols for the implementation of robotic rehabilitation treatments aimed at recovering upper limb function. Finally, it is important to emphasize that incorporating ML into robotic treatment has the potential to create a more individualized and efficient clinical practice, which would greatly benefit patients’ quality of life and the long-term viability of the healthcare system.

## Figures and Tables

**Table 1:** *p*-values and h-stats of the Jarque–Bera test.

Samples	H-Stats	*p*-Value
FMA T0	0	0.212
FMA T1	0	0.060
FAT T0	0	0.059
FAT T1	0	0.107
BI T0	0	0.500
BI T1	0	0.051

**Table 2:** *p*-values of the *t*-test between T0 and T1.

Clinical Scale	Samples	*p*-Value
FMA	T0 vs. T1	2.13 × 10^−6^
FAT	T0 vs. T1	7.53 × 10^−8^
BI	T0 vs. T1	1.33 × 10^−8^

**Table 3:** R^2^, RMSE, and MSE values for validation and test sets across various models.

Model	Clinical Scale	R^2^ Validation	RMSE Validation	MSE Validation
Bagged Ensemble of Decision Trees	FMA	0.79	0.368	0.226
	FAT	0.49	0.591	0.482
	BI	0.41	0.606	0.586
Gaussian Process	FMA	0.73	0.393	0.261
	FAT	0.41	0.633	0.579
	BI	0.74	0.416	0.255
Kernel regression	FMA	0.69	0.467	0.325
	FAT	0.46	0.696	0.633
	BI	0.62	0.548	0.385
Neural Network	FMA	0.56	0.786	0.832
	FAT	0.57	0.637	0.715
	BI	0.61	0.564	0.595

**Table 4:** *p*-values of *t*-tests between true values vs. predicted values.

Model	Clinical Scale	*p*-Value
Bagged Ensemble of Decision Trees	FMA	0.507
	FAT	0.856
	BI	0.802
Gaussian Process	FMA	0.955
	FAT	0.830
	BI	0.549
Kernel Regression	FMA	0.886
	FAT	0.959
	BI	0.845
Neural Network	FMA	0.713
	FAT	0.895
	BI	0.326

## Data Availability

The data presented in this study are available upon request from the corresponding author. The data are not publicly available due to privacy issues.
